# Isolation and molecular characterization of the *hemagglutinin* gene of H9N2 avian influenza viruses from poultry in Java, Indonesia

**DOI:** 10.5455/javar.2021.h530

**Published:** 2021-09-19

**Authors:** Christian Marco Hadi Nugroho, Otto Sahat Martua Silaen, Ryan Septa Kurnia, Retno Damajanti Soejoedono, Okti Nadia Poetri, Amin Soebandrio

**Affiliations:** 1 Doctoral Program Biomedical Science, Faculty of Medicine, University of Indonesia, Jakarta, Indonesia; 2 Animal Health Diagnostic Unit, PT Medika Satwa Laboratories, Bogor, Indonesia; 3 Department of Animal Diseases and Public Health, Faculty of Veterinary Medicine, IPB University, Bogor, Indonesia; 4 Eijkman Institute for Molecular Biology, Jakarta, Indonesia; 5 Department of Microbiology, Faculty of Medicine, University of Indonesia, Jakarta, Indonesia

**Keywords:** Avian influenza, HA gene, layer, phylogenetic analysis, sequencing, H9N2

## Abstract

**Objective:**

The avian influenza virus (AIV) subtype H9N2 circulating in Indonesia has raised increasing concern about its impact on poultry and its public health risks. In this study, the H9N2 virus from chicken poultry farms in Java was isolated and characterized molecularly.

**Materials and Methods:**

Thirty-three pooled samples of chicken brain, cloacal swab, trachea, and oviduct were taken from multiple chickens infected with AIV in five regions of Java, Indonesia. The samples were isolated from specific pathogenic-free embryonated eggs that were 9 days old. Reverse transcription polymerase chain reaction and sequencing were used to identify H9N2 viruses.

**Results:**

This study was successful in detecting and characterizing 13 H9N2 isolates. The sequencing analysis of *hemagglutinin* genes revealed a 96.9%–98.8% similarity to the H9N2 AIV isolated from Vietnam in 2014 (A/muscovy duck/Vietnam/LBM719/2014). According to the phylogenetic analysis, all recent H9N2 viruses were members of the lineage Y280 and clade h9.4.2.5. Nine of the H9N2 isolates studied showed PSKSSR↓GLF motifs at the cleavage site, while four had PSKSSR↓GLF. Notably, all contemporary viruses have leucine (L) at position 216 in the receptor-binding region, indicating that the virus can interact with a human-like receptor.

**Conclusion:**

This study described the features of recent H9N2 viruses spreading in Java’s poultry industry. Additionally, H9N2 infection prevention and management must be implemented to avoid the occurrence of virus mutations in the Indonesian poultry industry.

## Introduction

Since the early 1980s, Indonesia’s poultry sector has risen dramatically. The poultry industry in Indonesia is projected to be worth more than 34 billion US dollars. According to CASARED, there are approximately 67.9 million birds on 99,000 farms in Indonesia that use a commercially oriented production method [1,2].

In the last two decades, avian influenza virus (AIV) infection has become a common problem for the global poultry industry [3,4], including Indonesia. These viruses are categorized into two groups. The first group is an highly pathogenic AIV, for example, the H5N1 subtype which can cause mortality up to 100%. This subtype has become a major concern for human and animal health in Indonesia, especially the entire province of Java, since 2003 [5], and vaccinations have been routinely administered against this subtype. The second group was infected with various types of domestic and wild birds with mild to moderate infection called low pathogenic avian influenza virus (LPAIV) [6]. H9N2 belongs to this group and causes significant economic losses associated with increased mortality and decreased egg production [7]. Coinfection of bacteria such as *Mycoplasma gallisepticum* and *Escherichia coli* or other infections from other viruses such as infectious bronchitis (IB) and newcastle disease (ND) can increase the mortality of chickens infected with H9N2 [8]. Currently, this subtype is endemic to poultry in several African, Asian, and Middle Eastern countries [9].

There is a dearth of evidence on the characteristics of circulating AIVs subtype H9. The bulk of these virus sequences are classified as G1 (clade h9.4.1) or Y280 (h9.4.2 of clade). The h9.4.1 lineage includes viruses from India, Iran, Israel, and Pakistan, whereas the h9.4.2 lineage had all strains found in China. Prior to 2007, H9N2 was classified as clade h9.4.2.1–h9.4.2.4. After 2010, the clade H9.4.2.5 represented by Guangxi/55/2005 became well established and began spreading rapidly throughout the country. Furthermore, all h9.4.2.5 viruses discovered between 2013 and 2015 possessed human-like receptor specificity, implying the possibility of cross-species transmission [10]. 

Based on the duck sera’s susceptibility to recombinant *hemagglutinins* and neuraminidases in indirect enzyme-linked immunosorbent assay and dot blot studies, the H9N2 subtype was believed to exist in Java, Indonesia, duck population [11]. This is the first report of H9N2 outbreaks in Java, and surveillance will be required to confirm the existence of H9N2 subtypes via virus isolation and molecular characterization. In this work, we detected and examined H9N2 viruses from several regions of Java. 

## Materials and Methods

The study was carried out in April 2017–March 2018. The materials and methods used in this study are describe below.

### Ethical approval

The current study follows the requirements established by the Indonesian Law on Animal Health Research (UU/18/2009, article 80. Ethical approval was not required for this investigation because no live animals were used.

### Samples

A total of 33 commercial poultry chickens were sampled in 5 provinces of Java, Indonesia (Banten, *n* = 4; Central Java, *n* = 2; East Java, *n* = 9; West Java, *n* = 17; and Yogyakarta, *n* = 1) with a history of widespread depression, respiratory and neurological disorders, and decreased egg production. The poultry flocks comprised birds ranging in age from 25 to 79 weeks. Three moribunds or dead hens were randomly selected from each group and brought to the laboratory. The chicken brain, cloacal swab, trachea, and oviduct were taken and pooled for virus isolation. The samples collected are listed in Table 1.

### Virus isolation

Propagation of the sample was carried out according to conventional laboratory protocols. The swab or tissue was suspended in sterile phosphate buffer saline with penicillin (200 μg/ml) and streptomycin (100 μg/ml) in a 2:10–20 ratio and centrifuged at 1,000× *g* for 10 min at 4°C. The supernatant was filtered via a 0.22 μm membrane filter and inoculated into 9-day-old specific pathogenic-free (SPF) embryonated eggs via the allantoic route. The eggs were incubated at 37°C for 48–72 h and daily embryo death was monitored. Eggs that died before 24 h were discarded because the virus considered was non-specific. Harvested allantoic fluid was used in the hemagglutination (HA) experiment. Three serial passages were conducted prior to the selection of negative samples [12].

### Detection of viral RNA using reverse transcription-polymerase chain reaction (RT-PCR) test

Allantoic fluid was extracted using a total ribonucleic acid (RNA) mini kit (Geneaid, Taiwan). MyTaqTM OneStep RT-PCR kit (Bioline^®^, Taunton) was used to test the RNA for H9N2 viruses by RT-PCR utilizing H9 and N2-specific primers (Table 2). The following thermal profile was used to amplify the genes: 48°C for 20 min, 95°C for 2 min, followed by 40 cycles of 95°C for 10 sec, 52°C for 10 sec, and 72°C for 2 min. At the conclusion of the amplification, the final stage was carried out at 72°C for 10 min. Electrophoresis was used to visualize the amplified samples. The usual size marker is 100 bp (Geneaid, Taipei, Taiwan). Observations were conducted with the aid of a UV transilluminator. Additionally, real-time polymerase chain reaction (RT-PCR) assays for H5N1, Newcastle disease virus (NDV), and infectious bronchitis virus (IBV) were performed [17]. 

### Sequencing

Following good RT-PCR results for the H9 gene, the HA gene was amplified and sequenced using the MyTaq One-Step RT-PCR kit (Bioline) with the HA9-F and HA9-R primers (Table 2). Electrophoresis was used to separate the PCR products, and the desired band was purified for sequencing (by First BASE Laboratories Sdn Bhd, Malaysia). Determination of the nucleotide and amino acid sequences for the HA gene from a recently isolated strain using Bioedit v.7 and alignment with ClustalW. MEGA 7 was used to create the phylogenetic tree of contemporary viruses using the neighbor-joining method [18] with 1,000 alignment repeats [19]. Clades were defined using the genetic distance between members and the topology of the phylogenetic tree. We studied the molecular properties of amino acid sequence derivation from 13 nucleotide sequences. The nucleotide sequences of *hemagglutinin* genes were compared between the isolates from this investigation, isolated studies with prototype H9N2 strains, and isolated studies with H9N2 subtype vaccine strain China (chicken/Guangdong/SS/94, chicken/Shanghai/F/98, and chicken/Shandong/6/96).

**Table 1.** Collected samples for identification of AIV H9N2 in five provinces of Java, Indonesia.

Name of sample	District	Province	Age (weeks)	Collection date	Population
Chicken/Banten-01/17	Tangerang	Banten	64	20-Apr-17	>3,000
Chicken/Banten-02/17	Tangerang	Banten	56	20-Apr-17	>3,000
Chicken/WestJava-01/17	Cianjur	West Java	26	24-Apr-17	>5,000
Chicken/Banten-03/17	Tangerang	Banten	35	29-Apr-17	>3,000
Chicken/WestJava-02/17	Bogor	West Java	49	03-May-17	>4,000
Chicken/WestJava-03/17	Sukabumi	West Java	45	03-May-17	>10,000
Chicken/WestJava-04/17	Bogor	West Java	38	21-May-17	>3,000
Chicken/WestJava-05/17	Bogor	West Java	38	21-May-17	>3,000
Chicken/WestJava-06/17	Bogor	West Java	35	21-May-17	>3,000
Chicken/WestJava-07/17	Sukabumi	West Java	54	15-Jun-17	>7,000
Chicken/WestJava-08/17	Sukabumi	West Java	50	15-Jun-17	>7,000
Chicken/EastJava-01/17	Situbondo	East Java	33	16-Jun-17	>3,000
Chicken/ EastJava-02/17	Kediri	East Java	64	01-Jul-17	>3,000
Chicken/WestJava-09/17	Subang	West Java	25	05-Jul-17	>6,000
Chicken/ WestJava-10/17	Bogor	West Java	52	21-Jul-17	>4,000
Chicken/EastJava-03/17	Surabaya	East Java	48	22-Jul-17	>3,000
Chicken/EastJava-04/17	Situbondo	East Java	44	03-Aug-17	>2,500
Chicken/EastJava-05/17	Situbondo	East Java	52	03-Aug-17	>2,500
Chicken/WestJava-11/17	Subang	West Java	64	19-Aug-17	>8,000
Chicken/WestJava-12/17	Cianjur	West Java	54	21-Aug-17	>10,000
Chicken/EastJava-06/17	Situbondo	East Java	52	30-Aug-17	>3,000
Chicken/EastJava-07/17	Situbondo	East Java	72	30-Aug-17	>3,000
Chicken/EastJava-08/17	Situbondo	East Java	47	30-Aug-17	>3,000
Chicken/WestJava-13/17	Subang	West Java	41	01-Sep-17	>5,000
Chicken/WestJava-14/17	Subang	West Java	49	01-Sep-17	>5,000
Chicken/WestJava-15/17	Subang	West Java	36	01-Sep-17	>5,000
Chicken/Banten-04/17	Tangerang	Banten	31	03-Sep-17	>3,000
Chicken/EastJava-09/17	Blitar	East Java	36	05-Sep-17	>6,000
Chicken/Yogyakarta-01/17	Bantul	Yogyakarta	79	19-Sep-17	>3,000
Chicken/WestJava-16/17	Sukabumi	West Java	30	10-Oct-17	>6,000
Chicken/WestJava-17/17	Sukabumi	West Java	30	10-Oct-17	>6,000
Chicken/CentralJava-01/17	Surakarta	Central Java	26	17-Oct-17	>2,500
Chicken/CentralJava-02/17	Semarang	Central Java	29	21-Oct-17	>3,000

This study investigated several critical areas, including receptor-binding sites (RBS) on the left edge of the binding pocket, the right edge of the binding pocket, and the cleavage site. Additionally, certain amino acid residues (54, 80, 106, 109, 113, 123, 125, 129, 130, 135, 137, 146, 147, 149, 150, 152, 164, 165, 178, 179, 182, 183, 188, 189, 194, and 216) were evaluated for their association with H9N2 virus antigenicity. The amino acid sequences of the HA genes from the 13 most recent H9N2 viruses have been uploaded to http://www.cbs.dtu.dk/services/NetNGlyc/. to analyze probable N-glycosylation sites.

## Results

### Isolation and molecular identification of recent viruses

**Figure 1.**
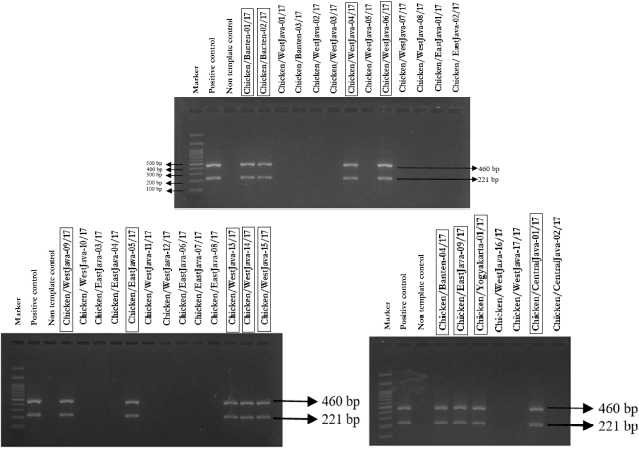
Visualization of the PCR product for the H9 (221 bp) and N2 (460 bp) genes of recent samples on an agarose gel. Electrophoresis results showed that from 33 research samples only 13 samples were positive for the H9 and N2 genes.

The result revealed that 18 samples were HA-positive after three passaged in embryonated chicken eggs with mean log 2, 4, and 8 HA units (Table 3), while the findings of RT-PCR showed only 13 samples were positive for H9N2 AIV (Fig. 1). Six of them were single infections of H9N2 AIV, while the seven positive samples were multiple infections by many coinfected viruses such as H5N1 (15.4%), NDV (38.5%), or IBV (7.6%). (7.6%). Regarding the distribution of positive H9N2 cases across different months, we observed that two samples (15.4%) were positive in April, two (15.4%) in May, one (7.7%) in July, one (7.7%) on August, six (48.2%) in September, and one (7.7%) in October 2017. 

**Table 2.** Oligonucleotide primers used for the detection of influenza viruses (H5 and H9), NDV, and IBV by RT-PCR assay.

Target gene fragment	Primer	Sequence (5′-3′)	Amplicon size (base pair)	Reference
H9	H9-F	ATCGGCTGTTAATGGAATGTGTT	221	[13]
	H9-R	TGGGCGTCTTGAATAGGGTAA		
N2	N2-F	CTCCAATAGACCCGTACTAT	460	[14]
	N2-R	CCTGAAGTCCCACAAAATAC		
H5	H5-F	ACAAAGCTCTATCAAAACCCAAC	499	[13]
	H5-R	TACCCATACCAACCATCTACCAT		
ND	ND-F	TGGAGCCAAACCGCGCACCTGCGG	766	[15]
	ND-R	GAGGATGTTGGCAGCAT		
IB	IB-F	GCTTTT GAG CCT AGC GTT	149	[16]
	IB-R	GCCATGTTGTCACTGTCTATT		
HA	HA9-F	CAAGATGGAAGTAGTATCACT	1,683	[10]
	HA9-R	TTGCCAATTATATACAAATGT		

### Sequence and phylogenetic analysis

**Figure 2.**
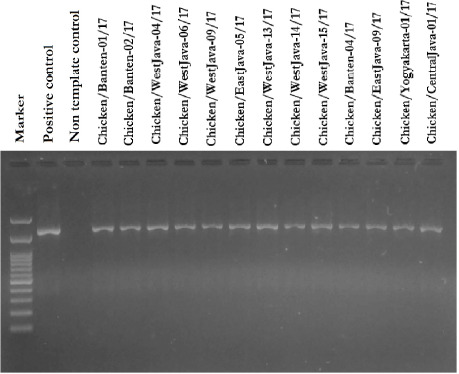
Visualization of PCR products on the HA gene of 13 H9N2 positive isolates on an agarose gel. Electrophoresis results showed that all isolates of VAI H9N2 were successfully amplified with a size of 1,683 bp.

**Figure 3.**
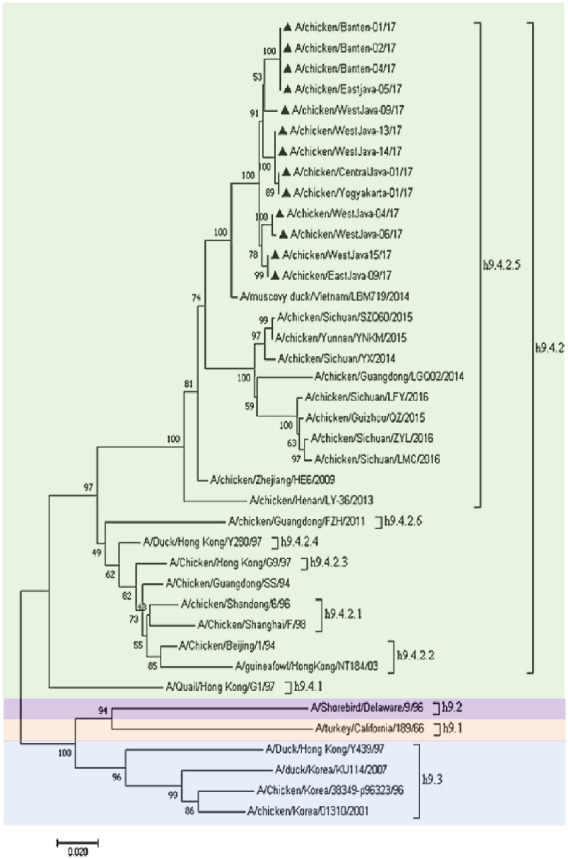
Phylogenetic tree of the HA gene of AIV H9N2 viruses. Recent Indonesian H9N2 viruses used in this study are represented with ▲. The region of HA was analyzed using MEGA version 7. The neighbor-joining bootstrap analysis (1,000 replicates) used the maximum composite likelihood method.

The RT-PCR experiment detected the presence of a complete HA gene with an amplicon size of 1,683 bp in the allantoic fluid (Fig. 2). The HA gene’s 13 most recent nucleotide sequences were deposited in GenBank with accession numbers MG957200, MG957201, MG957202, MG957203, MG957204, MG957205, MG957206, MG957207, MG957208, MG957209, MG957210, MG957211, and MG957212. A phylogenetic tree analysis based on the HA gene was created on the sequences of 13 recent isolates in our investigation and prior AIV H9N2 viruses [20]. Phylogenetic analysis revealed that all recent viruses belonged to clade h9.4.2.5 (Fig. 3), and a comparison of the 13 isolates’ HA sequences at the nucleotide level revealed 96.2%–100% identity with the lineage Y280 reference strain (Table 4). When compared to Chinese strains (Guangdong/SS/94, Shandong/6/96, and Shanghai/F/98), a reduced similarity rate (86.6%–92.3%) at the nucleotide level was identified. The present isolates had a nucleotide similarity of 96.9%–98.8% to the most recently circulating strains in Vietnam (muscovy duck/Vietnam/LBM719/2014) and China (chicken/Henan/LY-36/2013 and chicken/Zhejiang/HE6/2009). 

### Analysis of deduced amino acid sequences

The characteristics of LPAIV seen from the sequence of HA segments reveal a monobasic amino acid motif at the cleavage site (PSRSSR↓GLF) in 9 out of 13 study isolates, while 4 other isolates, A/chicken/Banten-01/17 (Accession No. MG957200), A/chicken/Banten-02/17 (Accession No. MG957201), A/chicken/Banten-04/17 (Accession No. MG957202), and A/chicken/EastJava-05/17 (Accession No. MG957210), showed a non-synonymous substitution of the amino acid (R317K) at this cleavage site to make PSKSSR↓GLF. All isolates in the present study showed H173N and Q217M mutations at receptor-binding sites while comparing the reference strains in GenBank. The E180A substitution was found in only five of our isolates, while the remaining seven isolates contain the E180T substitution (Table 5). 

**Figure 4.**
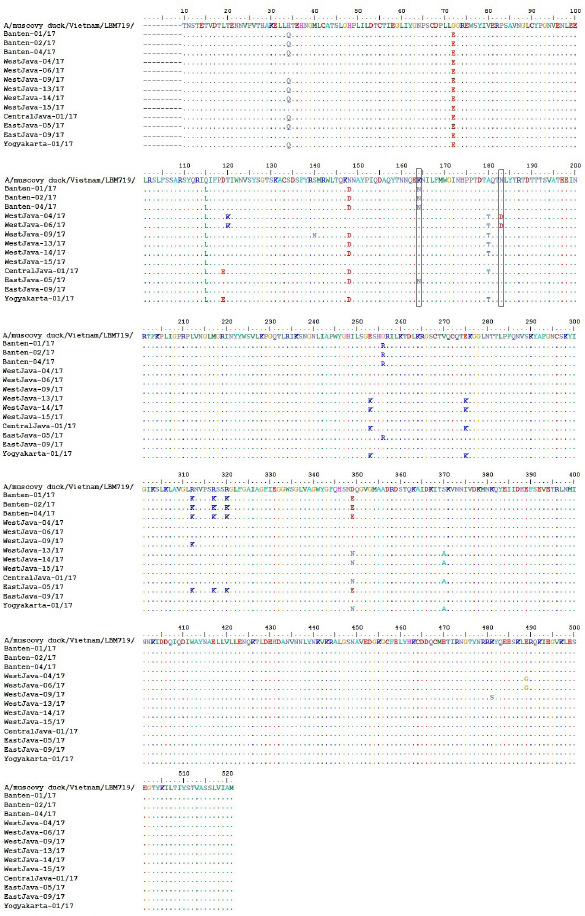
Multiple alignments of HA amino acid sequences in the field strains. Non-synonymous amino acid substitutions K164N and N183D had occurred in some isolates (in the black square).

The antigenic epitope of HA of H9N2 is mapped in two parts: site I and site II [21]. The antigenic site of the HA gene in all isolates obtained the same site I motif on the position residues of 125, 147, and 152, respectively, serine (S), lysine (K), and proline (P). While in site II, there are two kinds of motives at positions 135, 183, and 216. The first motif is aspartic acid (D), asparagine (N), and leucine (L), owned by 11 isolates in this study (Table 5). Unlike the others, N183D substitution occurs in two isolates from West Java, namely A/chicken/WestJava-04/17 (Accession No. MG957203) and A/chicken/WestJava-06/17 (Accession No. MG957204). One non-synonymous amino acid substitution (K164N) occurred in A/chicken/Banten-01/17 (Accession No. MG957200), A/chicken/Banten-02/17 (Accession No. MG957201), A/chicken/Banten-04/17 (Accession No. MG957202), and A/chicken/EastJava-05/17 (Accession No. MG957210) (Fig. 4).

**Table 3.** Results of isolation and identification using RT-PCR.

Name of Sample	HA test (HAU)	RT-PCR			
		H9N2	H5	NDV	IBV
Chicken/Banten-01/17	2^8^	**+**	-	-	-
Chicken/Banten-02/17	2^5^	**+**	-	+	-
Chicken/WestJava-01/17	2^8^	**-**	+	-	-
Chicken/Banten-03/17	-	**-**	-	-	-
Chicken/WestJava-02/17	2^5^	**-**	-	+	-
Chicken/WestJava-03/17	-	**-**	-	-	-
Chicken/WestJava-04/17	2^7^	**+**	-	-	-
Chicken/WestJava-05/17	-	**-**	-	-	-
Chicken/WestJava-06/17	2^4^	**+**	-	-	+
Chicken/WestJava-07/17	-	**-**	-	-	-
Chicken/WestJava-08/17	-	**-**	-	-	-
Chicken/EastJava-01/17	-	**-**	-	-	-
Chicken/ EastJava-02/17	-	**-**	-	-	-
Chicken/WestJava-09/17	2^4^	**+**	+	-	-
Chicken/ WestJava-10/17	2^5^	**-**	-	+	-
Chicken/EastJava-03/17	-	**-**	-	-	-
Chicken/EastJava-04/17	-	**-**	-	-	-
Chicken/EastJava-05/17	2^7^	**+**	-	+	-
Chicken/WestJava-11/17	-	**-**	-	-	-
Chicken/WestJava-12/17	2^8^	**-**	-	+	-
Chicken/EastJava-06/17	-	**-**	-	-	-
Chicken/EastJava-07/17	2^4^	**-**	-	+	-
Chicken/EastJava-08/17	-	**-**	-	-	-
Chicken/WestJava-13/17	2^6^	**+**	-	+	-
Chicken/WestJava-14/17	2^7^	**+**	-	-	-
Chicken/WestJava-15/17	2^5^	**+**	+	-	-
Chicken/Banten-04/17	2^8^	**+**	-	-	-
Chicken/EastJava-09/17	2^5^	**+**	-	-	-
Chicken/Yogyakarta-01/17	2^7^	**+**	-	+	-
Chicken/WestJava-16/17	-	**-**	-	-	-
Chicken/WestJava-17/17	-	**-**	-	-	-
Chicken/CentralJava-01/17	2^8^	**+**	-	+	-
Chicken/CentralJava-02/17	-	**-**	-	-	-

The potential glycosylation site of the HA gene analysis from 13 study isolates revealed 6 sites with N-X-T / S motif (where X is any amino acid except proline) in the HA1 gene section with positions in 11–13 (NST), 123–125 (NVS), 200–202 (NRT), 280–282 (NTT), 287–289 (NVS), and 295–297 (NCS) (Table 6).

## Discussion

Detection of H5N1 AIV from H9N2 positive cases indicated continuous co-circulation of the two subtypes in commercial chicken flocks. These study results agreed with previous studies [22] and provided a possible explanation for increased infectivity of H9N2 AIV over time due to genetic reassortment with the H5N1 subtype [23,24]. 

**Table 4.** Comparison of nucleotide sequence similarity from the 13 study isolates with several other H9N2 subtype isolates available in GenBank (%).

		13 recent isolates	Clade				
			h9.1^a^	h9.2^b^	h9.3^c^	h9.4.1^d^	h9.4.2^e^
13 recent isolates		96.2–100					
Clade	h9.1^a^	68.9–71.6					
	h9.2^b^	71–73.6	76				
	h9.3^c^	72.6–75.1	79.2	77.6			
	h9.4.1^d^	77.8–80.3	75.1	67.6	83.7		
	h9.4.2^e^	91.5–93.6	75.9	69.2	79.5	83.6	

a h9.1 represented by A/turkey/California/189/66.

b h9.2 represented by A/shorebird/Delaware/9/96/189/66.

c h9.3 represented by A/duck/Hong_Kong/Y439/97.

d h9.4.1 represented by A/quail/Hong_Kong/G1/97.

e 9.4.2 represented by A/duck/Hong_Kong/Y280/97.

**Table 5.** Receptor-binding pockets, cleavage sites, and antigenic site of several isolates of H9N2 studies compared with several different clade precursors (_*_).

Virus	Receptor-binding site	Left edge of the binding pocket	Right edge of the binding pocket	Cleavage site	Antigenic site		Accession Number
					Site I	Site II	
Turkey/California/189/66 (h9.1)	P.W.T.H.E.L.Y	N.D.Q.Q.G.R	G.T.S.R.A	P.A.V.S.S.R↓G.L.F	T.K.P	N.D.L	AF156390
Shorebird/Delaware/9/96 (h9.2)	P.W.T.H.E.L.Y	N.G.Q.Q.G.R	G.T.S.K.A	P.A.A.S.N.R↓G.L.F	T.K.P	N.D.L	AF156386
Duck/Hong_Kong/Y439/97 (h9.3)	P.W.T.H.E.L.Y	N.D.Q.Q.G.R	G.T.S.R.A	P.A.A.S.N.R↓G.L.F	T.K.P	N.N.L	AF156377
Quail/Hong_Kong/G1/97 (h9.4.1)	P.W.T.H.E.L.Y	N.D.L.Q.G.R	G.I.S.R.A	P.A.R.S.S.R↓G.L.F	T.K.P	G.N.L	AF156378
Duck/Hong_Kong/Y280/97 (h9.4.2)	P.W.T.N.T.L.Y	N.G.L.Q.M.R	G.T.S.K.A	P.A.R.S.S.R↓G.L.F	S.K.P	D.N.L	AF156376
Muscovy_duck/Vietnam/LBM719/2014 (h9.4.2.5)	P.W.T.N.A.L.Y	N.G.L.M.G.R	G.T.S.K.A	P.S.R.S.S.R↓G.L.F	S.K.P	D.N.L	LC028176
Chicken/Banten-01/17 (h9.4.2.5)	P.W.T.N.A.L.Y	N.G.L.M.G.R	G.T.S.K.A	P.S.K.S.S.R↓G.L.F	S.K.P	D.N.L	MG957200
Chicken/Banten-02/17 (h9.4.2.5)	P.W.T.N.A.L.Y	N.G.L.M.G.R	G.T.S.K.A	P.S.K.S.S.R↓G.L.F	S.K.P	D.N.L	MG957201
Chicken/Banten-04/17 (h9.4.2.5)	P.W.T.N.A.L.Y	N.G.L.M.G.R	G.T.S.K.A	P.S.K.S.S.R↓G.L.F	S.K.P	D.N.L	MG957202
Chicken/WestJava-04/17 (h9.4.2.5)	P.W.T.N.T.L.Y	N.G.L.M.G.R	G.T.S.K.A	P.S.R.S.S.R↓G.L.F	S.K.P	D.N.L	MG957203
Chicken/WestJava-06/17 (h9.4.2.5)	P.W.T.N.T.L.Y	N.G.L.M.G.R	G.T.S.K.A	P.S.R.S.S.R↓G.L.F	S.K.P	D.N.L	MG957204
Chicken/WestJava-09/17 (h9.4.2.5)	P.W.T.N.T.L.Y	N.G.L.M.G.R	G.T.S.K.A	P.S.R.S.S.R↓G.L.F	S.K.P	D.N.L	MG957205
Chicken/WestJava-13/17 (h9.4.2.5)	P.W.T.N.T.L.Y	N.G.L.M.G.R	G.T.S.K.A	P.S.R.S.S.R↓G.L.F	S.K.P	D.N.L	MG957206
Chicken/WestJava-14/17 (h9.4.2.5)	P.W.T.N.T.L.Y	N.G.L.M.G.R	G.T.S.K.A	P.S.R.S.S.R↓G.L.F	S.K.P	D.N.L	MG957207
Chicken/WestJava-15/17 (h9.4.2.5)	P.W.T.N.A.L.Y	N.G.L.M.G.R	G.T.S.K.A	P.S.R.S.S.R↓G.L.F	S.K.P	D.N.L	MG957208
Chicken/CentralJava-01/17 (h9.4.2.5)	P.W.T.N.T.L.Y	N.G.L.M.G.R	G.T.S.K.A	P.S.R.S.S.R↓G.L.F	S.K.P	D.N.L	MG957209
Chicken/EastJava-05/17 (h9.4.2.5)	P.W.T.N.A.L.Y	N.G.L.M.G.R	G.T.S.K.A	P.S.K.S.S.R↓G.L.F	S.K.P	D.N.L	MG957210
Chicken/EastJava-09/17 (h9.4.2.5)	P.W.T.N.A.L.Y	N.G.L.M.G.R	G.T.S.K.A	P.S.K.S.S.R↓G.L.F	S.K.P	D.N.L	MG957211
Chicken/Yogyakarta-01/17 (h9.4.2.5)	P.W.T.N.T.L.Y	N.G.L.M.G.R	G.T.S.K.A	P.S.K.S.S.R↓G.L.F	S.K.P	D.N.L	MG957212

(*) Positions 92, 143, 145, 173, 180, 184, and 185 (receptor binding site); 214–219 (left-edge of binding pocket, at position); 128–132 (right-edge of binding pocket); 315–323 (cleavage site); Antigenic site, divided into two namely site I at positions 125, 147 and 152; site II at positions 135, 183 and 216. Placement of residues according to H9 numbering.

**Table 6.** Comparison of the PGS in HA proteins of 13 H9N2 isolates.

Isolates	PGS								
	11–13	123–125	127–130	178–180	188–190	200–202	280–282	287–289	295–297
Chicken/Banten-01/17	N.S.T	N.V.S	–	–	–	N.R.T	N.T.T	N.V.S	N.C.S
Chicken/Banten-02/17	N.S.T	N.V.S	–	–	–	N.R.T	N.T.T	N.V.S	N.C.S
Chicken/Banten-04/17	N.S.T	N.V.S	–	–	–	N.R.T	N.T.T	N.V.S	N.C.S
Chicken/WestJava-04/17	N.S.T	N.V.S	–	–	–	N.R.T	N.T.T	N.V.S	N.C.S
Chicken/WestJava-06/17	N.S.T	N.V.S	–	–	–	N.R.T	N.T.T	N.V.S	N.C.S
Chicken/WestJava-09/17	N.S.T	N.V.S	–	–	–	N.R.T	N.T.T	N.V.S	N.C.S
Chicken/WestJava-13/17	N.S.T	N.V.S	–	–	–	N.R.T	N.T.T	N.V.S	N.C.S
Chicken/WestJava-14/17	N.S.T	N.V.S	–	–	–	N.R.T	N.T.T	N.V.S	N.C.S
Chicken/WestJava-15/17	N.S.T	N.V.S	–	–	–	N.R.T	N.T.T	N.V.S	N.C.S
Chicken/CentralJava-01/17	N.S.T	N.V.S	–	–	–	N.R.T	N.T.T	N.V.S	N.C.S
Chicken/EastJava-05/17	N.S.T	N.V.S	–	–	–	N.R.T	N.T.T	N.V.S	N.C.S
Chicken/EastJava-09/17	N.S.T	N.V.S	–	–	–	N.R.T	N.T.T	N.V.S	N.C.S
Chicken/Yogyakarta-01/17	N.S.T	N.V.S	–	–	–	N.R.T	N.T.T	N.V.S	N.C.S

The H9N2 virus has spread widely in poultry throughout the Asian region, providing the fact that the virus is potentially rapidly evolving with virulence rising [25,26,27]. It is similar to the report in Java. Lesser than a year ago, H9N2 AIV was found in all provinces of layer farms in Java. The presence of H9N2 in commercial farms of Java may indicate some defect in applying biosecurity [28].

The motif of the cleavage site of the recent isolate study was different from those of clades representative isolates. All H9N2 AIV fit the characteristic of LPAI. The proteases (such as trypsin), which are only secreted by the respiratory organs and intestines, will recognize a single arginine (R) residue [29].

The h9.4.2 was categorized as h9.4.2.1–h9.4.2.6 [30]. The recent H9N2 viruses isolated in this study belonged to h9.4.2.5 of the H9N2 clades. These results indicate that clade h9.4.2.5 dominated the predominant virus in Java.

While vaccination is the optimum technique for preventing AIV, earlier research indicates that inadequate protective immunization may result in an antigenic drift of the H9N2 virus [31]. To assess if a vaccination strain should be updated in response to the virus that is circulated in the field, an antigenic link between the commercial vaccine strain and the emerging recent virus should be evaluated [32].

The receptor-binding specificity was associated with amino acids in the RBS of HA proteins. In general, AIVs specifically recognize sialylα2,3-galactose receptors, while the human virus recognizes the sialyl-α2,6-galactose receptor [33]. Hemaglutinine proteins of H9N2 viruses isolated from chickens in a recent study had histidine (H), glutamic acid (E), glutamine (Q), and glutamine (Q) at 173, 180, 216, and 217 positions at the RBS [34]. The amino acid substitution in this study had previously appeared in Chinese isolates (A/chicken/Guangdong /LGQ02/2014). The presence of asparagine (N) at 173 positions is owned by the AIV subtype H9N2 belonging to clade h9.4.2, whereas methionine (M) at position 217 can only be encountered in clade h9.4.2.5. 

Leucine (L) at position 216 was found mainly in the HA protein of H2 and H3 viruses isolated from humans. In a previous study, Leucine (L) at 216 caused a van der Waals bond with the C-6 atom to form a sialyl-α2,6-galactose linkage. HA having leucine (L) at position 216 tends to bind to sialyl-α2,6-galactose, while Q in that position tends to bind to sialyl-α2,3-galactose. The substitution of Q to L in position 216 allows the H9N2 virus to replicate more efficiently in human cell culture [34]. The substitution of Q to L at position 216 is one of the genetic changes that occurs during the adaptation of avian influenza strains in pigs [35]. The existence of a backyard poultry system exacerbates the condition. Infected poultry without any clinical symptoms may increase the likelihood of exposure in humans [25].

The N183D mutation with Q216L at antigenic site II is involved in inhibiting viral interactions with epitope-specific antibodies and can result in mutant virus escape; both of these residues are identical to those in the most closely related vaccine. The 127 amino acid residue is another critical location in the HA H9N2 gene [21]. In a recent investigation, all H9N2 viruses from Java showed residual S127, which was identical to that of the H5 subtype [36]. Recent research [37] identified strains of H9 variations having the S127N mutation, which may increase the pathogenicity of SPF hens, while 13 isolates lacked the mutation.

In general, N-linked glycosylation has a substantial effect on the virus’ biological features. It may be impacted by the N-X-S/T-X amino acid structure [38]. Additionally, earlier research has demonstrated that alterations in glycosylation residues near HA protein cleavage sites might influence viral virulence [39]. Previous research has established that N-linked glycosylation of the HA protein has an effect on host innate immune system evasion and survival, as seen in human influenza virus, H1 and H3 subtypes [40]. Changes in the glycosylation status of the antigenic protein of the influenza virus HA region alter virus binding to antibodies, whereas changes in the glycosylation site at numerous places have no effect on the structure and function of HA [41]. There was a potential glycosylation site (PGS) within the antigenic area, NVS, between locations 123 and 125 in this study. Previous research with H1N1 viruses revealed that the presence of K123N mutations results in immune evasion via glycosylation [42]. 

## Conclusion

In five regions of Java, the H9N2 virus was isolated from chickens. The H9N2 virus that recently circulated in Java is classified as clade h9.4.2.5 and lineage Y280. Additionally, all recent viruses have a genetic link with viruses from China and Vietnam.

## List of Abbreviations

AIV: Avian influenza viruses, HA: Hemagglutination, HAU; Hemagglutination unit, IBV: Infectious bronchitis virus, LPAIV: Low pathogenic avian influenza virus, ND: Newcastle disease, PBS: Phosphate buffer saline, RBS: Receptor-binding site, RNA: Ribonucleic acid, RT-PCR: Reverse transcription polymerase chain reaction, SPF: Specific pathogenic-free.
